# Challenges with tracing patients on antiretroviral therapy who are late for clinic appointments in rural South Africa and recommendations for future practice

**DOI:** 10.1080/16549716.2020.1755115

**Published:** 2020-04-28

**Authors:** David Etoori, Alison Wringe, Jenny Renju, Chodziwadziwa Whiteson Kabudula, Francesc Xavier Gomez-Olive, Georges Reniers

**Affiliations:** a Department of Population Health, London School of Hygiene and Tropical Medicine, London, UK; b Department of Epidemiology and Biostatistics, Kilimanjaro Christian Medical University College, Moshi, Tanzania; c MRC/WITS Rural Public Health and Health Transitions Research Unit (Agincourt), School of Public Health, University of the Witwatersrand, Johannesburg, South Africa

**Keywords:** Loss to follow-up, tracing, HIV, engagement, retention

## Abstract

**Background**: It is common practice for HIV programmes to routinely trace patients who are late for a scheduled clinic visit to ensure continued care engagement. In South Africa, patients who are late for a scheduled visit are identified from clinic registers, and called by telephone up to three times by designated clinic staff, with home visits conducted for those who are unreachable by phone. It is important to understand outcomes among late patients in order to have accurate mortality data, identify defaulters to attempt to re-engage them into care, and have accurate estimates of patients still in care for planning purposes.

**Objective**: We conducted a study to assess whether tracing of HIV patients in clinics in rural north-eastern South Africa was implemented in line with national policies.

**Methods**: Thirty-three person-day of observations took place during multiple visits to eight facilities between October 2017 and January 2018 during which clinic tracing processes were captured. The facility level implementation processes were compared to the intended tracing process and gaps and challenges were identified.

**Results**: Challenges to implementing effective tracing procedures fell into three broad categories: i) facility-level barriers, ii) issues relating to data, documentation and record-keeping, and iii) challenges relating to the roles and responsibilities of the different actors in the tracing cascade.

We recommend improving linkages between clinics, improving record-keeping systems, and regular training of community health workers involved in tracing activities. Improved links between clinics would reduce the chance of patients being lost between clinics. Record-keeping systems could be improved through motivating health workers to take ownership of their data and training them on the importance of complete data. Finally, training of community health workers may improve sustained motivation, and improve their ability to respond appropriately to their clients’ needs.

**Conclusions**: Substantial investment in data infrastructure and healthcare staff training is needed to improve routine tracing.

## Background

At the end of 2017, it was estimated that 34.6 million adults aged 15 years and older were infected with HIV worldwide, 70% of whom resided in sub-Saharan Africa [1]. New treatment guidelines calling for immediate lifelong treatment for everybody testing positive for HIV (known as “Test and Treat”) resulted in 15.4 million individuals initiating antiretroviral therapy (ART) by the end of 2017, representing 60% of all people living with HIV (PLHIV) in sub-Saharan Africa [1]. By the end of 2015, South Africa had the largest ART programme in the world [2,3]. In 2016 South Africa adopted the ‘Test and Treat’ policy which translated to even more people being eligible for treatment [4,5]. By the end of 2018, an estimated 68% of the 7.2 million PLHIV in South Africa were on ART [1,6].

PLHIV who are taking lifelong ART who are late for scheduled clinic appointments are labelled as lost to follow-up (LTFU), a general term that amalgamates several possible outcomes including death, default, and self-transfer to another clinic [7–9]. Failure to account for the true outcomes of patients deemed LTFU leads to as much as five-fold underestimation of retention because silent (undocumented) transfers are not taken into account [10]. Similarly, default rates are over estimated as all patients that are LTFU are assumed to have stopped taking treatment [10,11]. Furthermore, if only deaths reported to the clinic are included in mortality estimates this results in them being underestimated. Inaccuracies in calculating the actual number of people alive and on ART has implications in the estimation of national ART coverage and corresponding ART programme budgets. Silent transfers can lead to double counting of the number of people who have ever initiated ART which could lead to overestimates of ART supplies needed, and over-estimates of ART programme coverage in national evaluations which could result in a reduced focus on reaching coverage targets [12]. Finally, misclassification of patients who are alive and on ART elsewhere as LTFU underestimates the impact of ART on mortality [11,13] which is an important statistic for programme monitoring as well as for informing HIV modelling and projections by UNAIDS [14–16].

Effective tracing programmes are likely to become increasingly important in the context of ‘Test and Treat’ strategy as more asymptomatic patients are initiating ART and may have higher rates of LTFU [17]. Tracing is effective at improving engagement with studies showing that as many as 86% of patients who had defaulted from care reengage in care following tracing [8,18,19] and that active tracing significantly reduces attrition [11,20]. Moreover, continued retention is crucial to mitigate the risk of development of resistance in patients who do not adhere correctly to ART [21–24]

Previous studies have documented some challenges related to tracing patients in HIV programmes in sub-Saharan Africa including organisational challenges, health worker shortages, and high costs which continue to limit the ability of HIV programmes to trace patients who are missing or LTFU [25,26], but few recommendations are made to mitigate these challenges.

This paper describes the challenges that were observed with routine tracing in the primary health care system in north-eastern South Africa, comparing policy to actual implementation. The observations were made whilst undertaking a study to consolidate routine tracing information and conduct supplementary tracing for PLHIV who were LTFU in a decentralised HIV treatment programme in the same setting. Recommendations to address these challenges are made to improve the implementation of tracing systems in this setting and beyond.

## Methods

### Setting

The clinics included in the broader tracing outcomes study were those serving the population of the Agincourt Health and Demographic Surveillance System (HDSS). The HDSS study area covers 475 km^2^ in Bushbuckridge, Mpumalanga province, north-eastern South Africa. Agincourt HDSS has been tracking demographic and health events (i.e. births, deaths and migration) in Tsonga or Shangaan people since 1992 [27,28]. By 2014, the population was approximately 115,000 people living in 17,000 households spread over 30 villages [28]. The study site is run under the auspices of the Medical Research Council (MRC)/Wits Rural Public Health and Health Transitions research unit, administrated by the School of Public Health at the University of Witwatersrand (WITS). Agincourt HDSS population is served by five primary health clinics and three secondary community health centres. Every HIV-positive patient has a clinical file that is established when they first register at the ART clinic and updated at each clinic visit. Following the clinic visit, visit-level information from the patient file is entered into the national electronic database, TIER.Net. Since 2014, Agincourt HDSS has undertaken an exercise called Point-of-contact Interactive Record Linkage (PIRL) [29] where chronic care patient visits in the clinics have been recorded and linked to the patient’s HDSS record, provided that they ever lived in the HDSS.

### Study design

The challenges and recommendations presented in this paper are based on 33 person days of observations in eight primary healthcare facilities (Table 1) in the Bushbuckridge sub-district between October 2017 and January 2018. Clinics were chosen because they were located within the HDSS. Each clinic was visited at least three times over this period to ascertain tracing outcomes for patients that were believed to be LTFU (more than 90 days late for a schedule clinic appointment). Briefly, this mixed methods study involved a comprehensive review of available clinic records documented through TIER.Net (the national electronic HIV patient monitoring database) and paper-based patient clinic files. We also consulted logbooks from Right-to-Care (RtC) and Home-Based Carers (HBC), two non-profit organisations that assist with tracing HIV patients LTFU through telephone calls and household visits respectively (Box 1).

**Box 1.** Description of the organisations that assist with tracing in Agincourt

Right-to-care: Founded in 2001 Non-profit organisation who provide prevention, care and treatment for HIV and associated diseased (tuberculosis, cervical cancer, and other STIs) Work with government and communities to find solutions to build and strengthen public health care In Agincourt, this constitutes assistance with tracing usually through assistance with telephone tracing Home-Based cares: Started in the late 1980s in rural villages of the Limpopo region Introduced as a way of improving healthcare practices to promote health through population sensitisation around aspects including child care, nutrition, and personal hygiene In recent years they have become more strucutred and are at the forefront of healthcare service delivery including delivery of treatment, care and support for people living with HIV In Agincourt they assist with healthcare promotion and physical tracing of people living with HIV who are late for their scheduled clinic visits

**Table 1.** Selected characteristics of the eight clinics that serve the study area

Clinic	Type	Number of HDSS residents 18+ years that initiated ART between 2014–2017	Right to care presence	Personnel consulted	Number of days of observations
Agincourt community health centre	Public	975	Yes	Ward Based Outreach Team (WBOT) nurse, Facility manager (Sister-in-charge), Home Based Carers, Right-to-Care linkage officer, community health worker	6
Belfast clinic	Public	582	Yes (started July 2017)	Data typist, Right-to-Care linkage officer, Home Based Carers	4
Bhubezi community health centre	Public (originally private, merged with Lillydale clinic a public facility, in 2016)	689	Yes	Home Based Carers, 2 Data typists, Right-to-Care linkage officer, Right-to-Care supervisor	5
Cunningmore clinic	Public	300	No (Linkage officer resigned in 2016)	Data typist, 2 staff nurses, Home Based Carers, Right-to-Care supervisor	3
Justicia clinic	Public	423	No	Data typist, Home Based Carers, staff nurse	4
Kildare clinic	Public	586	Yes	Facility manager, data typist, Right-to-Care linkage officer, Home Based Carers	5
Thulamahashe community health centre	Public	133	Attached (same linkage officer works in Belfast and Bhubezi)	Data typist, Right-to-Care linkage officer	3
Xanthia clinic	Public	235	Attached (same linkage officer works in Agincourt)	Data typist, Home Based Carers, Right-to-Care linkage officer	3

We then worked with these organisations to conduct a further home visit for all patients for whom routine tracing had not previously been undertaken, or for those without a definitive outcome after record review. Observations of how the tracing systems operated were captured in logbooks. Primary healthcare facility managers usually a sister-in-charge (nurse) were informally consulted for further clarification of how the implementation took place in practice. In cases where the sister-in-charge was unavailable, RtC officers, clinic data typists or other nurses were consulted. We also visited ten HBC organisations to document how their work intersected with that of the clinics’ tracing system. Additionally we reviewed one national policy document [30], which detailed how tracing should take place and had further conversations with other stakeholders and key actors.

### Data collection

As part of the comprehensive clinic record review, we collected data from TIER.Net and patient files for 1325 patients that met the LTFU criteria on 15 August 2017. Data included information on the patient’s treatment status (i.e. still in care, deceased, transferred out) and whether they had received a tracing intervention (typically a comment in their record about telephone or physical tracing). From RtC and HBC, we asked to view their tracing logs and collected data on any telephone or physical tracing interventions for each LTFU patient and the outcome of this tracing. Data were entered into a Microsoft access database. Discussions with HBCs centred around how physical tracing was performed in practice, and their interactions with clinic staff and patients.

In each clinic, through discussions with the staff, we identified the most knowledgeable person regarding each step and where possible asked for an explanation of how tracing typically occurred. We also asked to view all data collection instruments used in the tracing process including late patient lists and the tracing registers. Data were entered into an Excel spreadsheet.

### Definitions

Implementation of tracing was defined as being optimal if lists of late patients, tracing registers, telephone and physical tracing were used as intended in policy and well documented with clinic staff able to produce these instruments when requested. Tracing implementation was defined as ‘inconsistent’ if it did not align with policy or was not well documented. In the case of telephone and physical tracing inconsistency could also mean that these steps were not well documented, but we found evidence of telephone and physical tracing in patient files or TIER.Net. Tracing implementation was classified as ‘not observed’ where clinic staff could not produce these instruments when requested and we could not find evidence of any intervention in patient files, TIER.Net, or any documentation kept by RtC and HBC and was classified as ‘not done’ where clinic staff admitted that an intervention was not performed.

### Data analysis

Data from the clinic record review was exported to Stata 14 for analysis. We produced a checklist for each clinic to determine whether each tracing step was optimally used, inconsistent, not observed or not done. Data were then summarised using proportions and frequencies.

## Results

### Expected implementation of the routine tracing system

**Figure 1.**
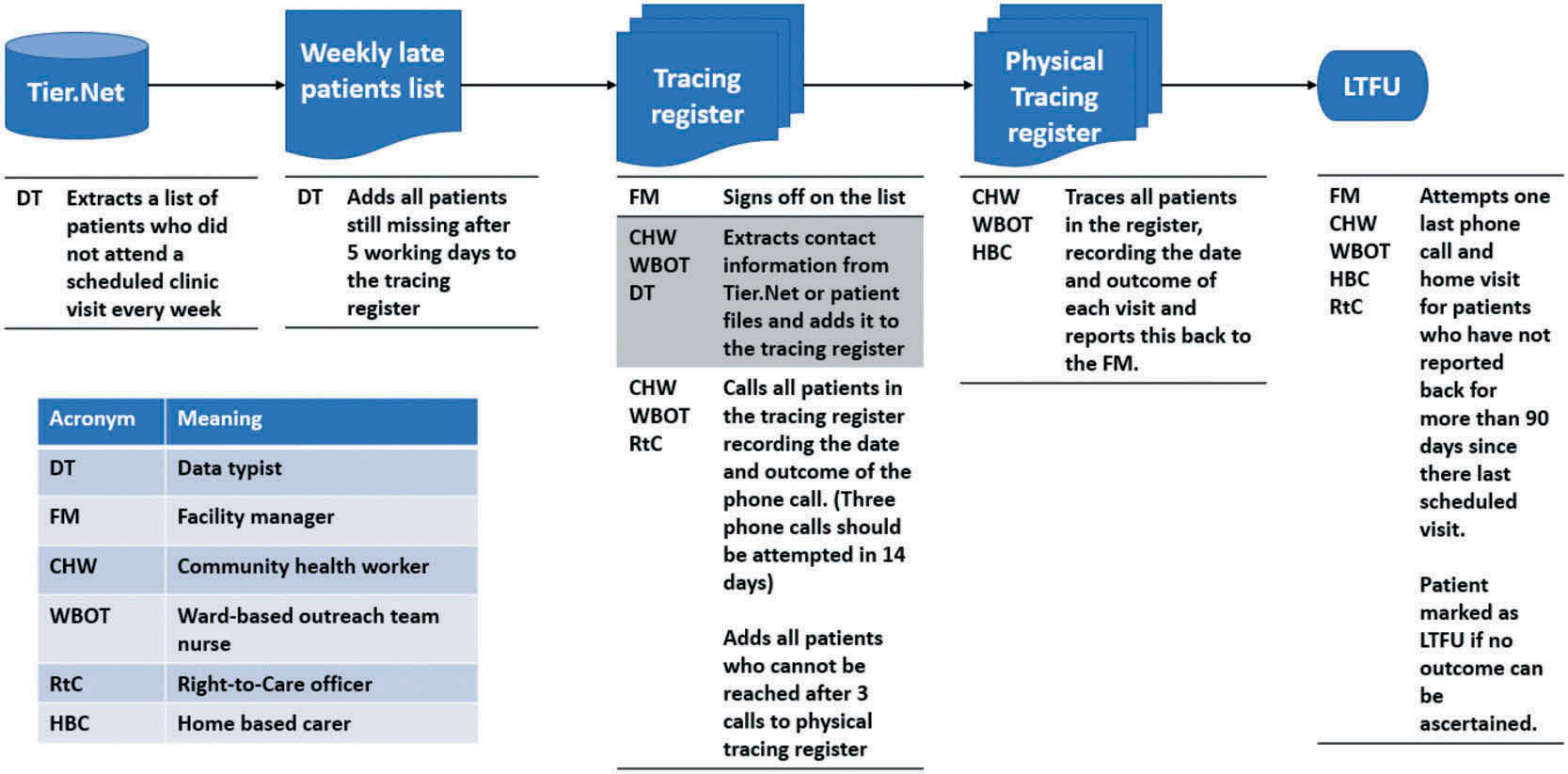
Tracing steps and personnel in charge of each step

In all of the clinics in this study setting LTFU was defined as being more than 90 days late for a scheduled appointment in accordance with national policy [30]. All health facilities routinely traced HIV patients who were LTFU (Figure 1). Each health facility manager is responsible for ensuring that there is a functioning paper-based or electronic appointment system such that clinical files for patients who are expected the following day are retrieved from the filing room. Files for patients who do not attend a scheduled visit should be kept aside for further action. A list of patients who did not attend a scheduled appointment should be generated every week, either through the facility’s appointment register or through querying the facility-level electronic database (TIER.Net). If a patient has not attended the facility within five working days to follow-up on a missed scheduled appointment, the patient’s name should be registered in the facility tracing register to be traced.

This list should be signed off by the facility manager and transferred to the person responsible for tracing patients, usually a designated nurse, community health worker (CHW) or the ward-based outreach team (WBOT) lead. The delegated nominee should extract contact information including addresses and telephone numbers (which should be updated at every clinic visit) of the individuals on the list (and their treatment supporter where available) from the patient files and enter this information into the facility tracing register. The facility telephone should then be used to contact all the individuals who were added to the tracing register that week, with the date the phone call was attempted and the outcome of the phone call, and patient outcomes recorded in the register when obtained. Three calls should be attempted within 14 days after each patient’s missed visit. Patients found to be alive, and who have not transferred to another clinic should be encouraged to return to treatment. Self-transfers should be further investigated usually through a phone call to the facility they have transferred to (Six facilities receive assistance with telephone tracing from RtC). The names of patients who cannot be reached after three attempts by phone should be transferred to a list of those to be traced through outreach and home visits. Patient consent for a home visit should be verified in the patient file before HBC attempt household visits.

WBOTs, CHWs and HBC linked to the facility should be involved to physically trace defaulters. Details of each home visit, including the outcome of the visit, should be reported to the facility manager. Each outreach tracing effort should be marked in the facility tracing register, indicating the date and outcome of the tracing visit.

Patients who have not reported back to the clinic for 90 days since their last scheduled visit and do not have a tracing outcome e.g. transferred out, died or stopped treatment are then in principle registered as LTFU. Before this entry is made, one more attempt at phoning or visiting the patient should be made. Information from this entire exercise should then be documented into the patients’ electronic record.

### Actual observed performance of the routine tracing system in the eight health facilities

**Figure 2.**
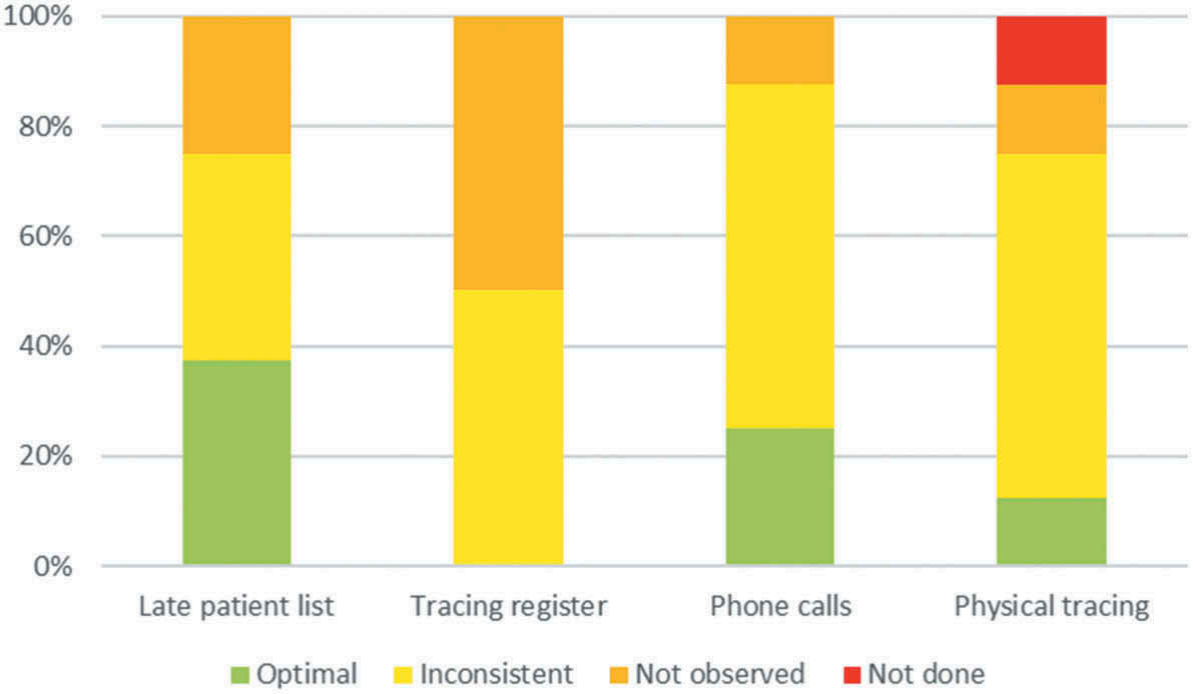
Performance on specific indicators from the tracing cascade for eight health facilities

Overall, none of the eight clinics had optimal performance with regards to any of the indicators (Figure 2). Optimal use of late patient lists was observed in 3 (37.5%) clinics, was inconsistent in 3 (37.5%) clinics and was not observed in 2 (25.0%) clinics. Similarly, tracing registers were inconsistently used in 4 (50.0%) clinics and not observed in 4 (50.0%) clinics. Phone calls were optimally used and consistently documented in 2 (25.0%) clinics, inconsistent in 5 (62.5%) clinics and not observed in 1 (12.5%) clinic. Finally, physical tracing was optimally used and documented in 1 (12.5%) clinic, inconsistent (usually due to poor documentation) in 5 (62.5%) clinics, not observed in 1 (12.5%) clinic, and was not done in 1 (12.5%) clinic. In both clinics, HBC and other community health workers were not engaged in the tracing procedures.

### Challenges identified in implementing the routine tracing system

Through the data collected, conversations with stakeholders and observations, we identified three major types of challenges to the optimal implementation of tracing in the eight facilities: facility-level barriers; challenges relating to data, documentation and record-keeping; and challenges relating to the roles of tracing personnel.

### Diversity in clinic procedures

Whilst all the clinics drew guidance on patient tracing procedures from the same national guidelines and documents, in practice, their implementation of these guidelines varied. For example, each clinic that conducted routine tracing employed a different filing system and process for generating patient identification (ID) numbers. In some cases, these were based on patient characteristics such as birth dates, whereas in others, they were generated using sequential numbers. In one clinic, two different patient ID systems had been implemented, and while one had been discontinued in favour of another, both types of patient IDs remained in operation. The way in which files were stored was also different across the clinics, with some of them cataloguing and indexing files while others did not have any discernible filing system. Tracing procedures were also not consistent between clinics with different actors involved, and in some cases, certain tracing steps were not performed.

Furthermore, each clinic used TIER.Net independently with no links between the different clinic databases. This meant that if patients chose to move between clinics there was no clear way of identifying them as patients who were already in care elsewhere and a new file was opened in the receiving clinic. Moreover, the limited communication between clinics affected the efficiency and coverage of tracing activities. For example, if patients resided outside the catchment area of the HBC organisations attached to the clinic they were using, they would not be physically traced, as this would need the involvement of HBC from another clinic.

### Data, documentation and record-keeping challenges

Weekly lists of patients late for a scheduled clinic visit were always manually generated from TIER.Net by data managers. We observed that this was prone to human error with some patients who should have been categorised as late for a scheduled appointment being missed completely and not receiving any tracing interventions. In five clinics, a total of fifty-three patients who had not visited the clinic for more than 90 days were recorded as being still in care and had been missed by the data manager when the LTFU lists were generated. Additionally, whilst national tracing guidelines indicate that every clinic must keep a facility tracing register for planning, monitoring and research purposes, in most clinics, these registers could not be found and appeared not to be part of routine tracing practice. Furthermore, both TIER.Net and patient files did not have a dedicated space or module for capturing tracing outcomes. In some clinics, these outcomes were sometimes entered into the comments section, but this was not consistently done. Patients in some differentiated care models, including adherence groups and fast-track treatment collection appointments visited their clinic less frequently and in some cases do not have to interact with clinic staff when they pick up refills. However, TIER.Net and patient files did not always reflect this information about patients. This meant that some patients were erroneously categorised as LTFU, whereas they were still in care through differentiated care models.

Whilst both RtC and HBC organisations were active patient tracing partners, they did not always keep records of their work. In one clinic, an HBC volunteer admitted that they traced patients, but did not document the tracing, likely due to a myriad of reasons including a lack of training, a lack of understanding of the importance of documenting their work, and stationery shortages. Also, standardised tracing forms were often unavailable, or unused in practice, and some HBC being apparently unaware of them. In some clinics, supplies of tracing forms were expected to be generated by photocopying an existing form, but the equipment to do this was not always available or functioning. This led to staff utilising logbooks or exercise books for record keeping, or in the worst case, not documenting their work at all. Impromptu logbooks often had incomplete data and in some cases, had been lost. HCWs struggled to accurately and consistently capture quality data as they prioritised their clinical work.

Additionally, tracing could only be performed if correct patient telephone numbers and addresses were available in their file. Also, for some patients the telephone number used by the clinic was only effective the first time as once patients recognised the clinic telephone number, they would not respond to any further calls received from it.

### Challenges relating to roles and responsibilities of personnel involved in the tracing system

The number of actors involved in routine tracing varied between clinics, and we observed duplication of work or transfer of tasks and responsibilities from one actor to another. For example, as the list of patients who are LTFU is generated from TIER.Net, it is the responsibility of the data manager to crosscheck the LTFU list with physical files to confirm that no patient visit was missed and not entered into the database. However, in one of the clinics, this duty had been shifted to the WBOT nurse, meaning she dedicated less time to her clinical work. In some clinics, the different actors were not aware of each other’s roles, meaning that work was duplicated, or that in other cases some patients did not receive all the tracing interventions because some actors did not receive their information. In one clinic, the HBC and RtC personnel had never communicated, culminating in some patients never being discussed nor physically traced. Also, whereas tracing organisations said they informed data managers about tracing done, this was not always reflected in TIER.Net.

Finally, we heard of various challenges within the different home-based care organisations. Many of the HBC felt that they were under-funded, and that because of this HBC staff worked on a voluntary basis, only receiving a small stipend from the health department and some non-monetary incentives. Some expressed a loss of motivation to do their work which was fostered by their dissatisfaction with this system. Furthermore, HBC had not received formal training which could in some way explain the patients concerns over conduct, particularly in terms of maintaining confidentiality when they attempted to trace them. Further issues included cases whereby the HBC asked family members or friends of the patient why they had not attended the clinic, thereby inadvertently disclosing their HIV status. However, where HBC were active and motivated, they were more likely to become aware of a patient missing an appointment before they were contacted by the clinic. In one clinic, HBCs routinely visited patients ranking them by a colour system as stable (green – seen every 1–2 months) or volatile (yellow – seen fortnightly or red – seen weekly). If they suspected that a patient was LTFU they then confirmed this at the clinic, later going back to try to reengage the patient into care. This was a more practical system, relying less on communication between actors and less prone to errors due to miscommunication.

## Recommendations for future practice

We found that most clinics had a system in place to identify and trace patients that had missed a scheduled clinic appointment. However, in practice, these procedures were heterogeneous and often led to gaps, leakages and missed opportunities within the tracing system. Ultimately this meant that tracing efforts were inconsistent and probably inefficient. We identified three main areas of challenges with routine tracing activities related to facility-level barriers; data, documentation and record-keeping challenges; and the roles and responsibilities of the different actors.

### Improved linkages between clinics

Each clinic in Agincourt HDSS has a catchment area that includes one or more villages within its vicinity. Home-based carers from the villages in this catchment area are attached to the clinic. However, many patients choose to initiate treatment at a clinic that does not correspond to their home village [31–33]. In such instances, the current tracing strategy is significantly constrained due to a lack of linkages between clinics. Such challenges are further perpetuated by the way in which clinics are driven to achieve national targets. National clinic evaluation meetings often present clinic targets in a way that fosters competition amongst clinics, putting unnecessary pressure on HCW to meet these targets [34]. This competition might improve some targets (e.g. number of patients tested or initiated on ART) but undermine the general effectiveness of tracing endeavours.

Clinics should be encouraged to coordinate efforts working together to trace patients. In cases where a patient is under the jurisdiction of HBC not attached to a given clinic, there should be procedures that allow for the clinic to communicate with the HBCs to facilitate tracing. This would have to be coupled with significant patient education and sensitisation about the system to make sure they do not feel that their confidentiality has been breached. Patients should also be sensitised on the importance of declaring prior medical history when presenting at a new facility. Long term, linkages between the different clinic electronic recording systems and better implementation of unique patient identification numbers would also help to identify silent transfers.

### Enhancements of recording systems

Data quality issues including incompleteness have been reported as a problem for tracing in other HIV treatment programmes [9]. Studies suggest that data quality issues can be attributed to limited engagement of HCWs with the data for their own planning, research, and monitoring and evaluation purposes [35] as well as a lack of resources (e.g. logbooks). Insufficient training of HCWs responsible for recording information and inadequate auditing procedures to ensure that incomplete records are identified and rectified in a timely manner further exacerbates this issue [35]. Healthcare workers need to balance different priorities with data entry often seen as less important.

Large investments have been made in establishing electronic medical records at all the clinics and each clinic has a dedicated data typist for Tier.Net, solely responsible for electronic data entry. However, the system still exists in an ad-hoc fashion (the data typist copying information from paper records) and is not fully integrated into medical practice (i.e. healthcare providers directly entering information into the system). Efforts are needed to fully integrate data entry into good clinical practice and clinic staff should be trained on the importance of recording all patients’ outcomes. Furthermore, efforts should be made to motivate HCWs to take ownership of, and utilise their data for monitoring and evaluation. Whilst a dedicated module for tracing might be unrealistic in the near future, additional training on basic data analysis, provision of mentorship opportunities, as well as training on auditing records could lead to improvements in data completeness. Development of standard operating procedures for data entry and training on these could further improve data completeness.

### Training for lay community healthcare workers

The inadequate healthcare workforce in sub-Saharan Africa has been well documented [36,37], and task shifting has been widely recommended and implemented as an efficient and sustainable strategy to expand HIV treatment programmes in this setting [38,39]. However, we observed that the voluntary nature of HBCs, and the non-transparent recruitment strategies [40] can be problematic. These cadres are expected to be part of the health system, but not necessarily part of its organisation. They have shorter training than professional healthcare workers, and they require minimal qualifications [39]. We observed concerns around their ability to handle confidential information. Over thirty years ago, Walt [41] identified several challenges to the success of HBC including lack of remuneration, insufficient training, poor management, and lack of supervision and logistical support which we found to still be prevalent in this setting.

HBCs should receive specific training in conducting tracing activities as this has been associated with sustained motivation, as well as with improving their ability to respond appropriately to their clients’ needs [42] and could reduce issues related to breaches of confidentiality which undermine tracing efforts. Training could also contribute to furthering the legitimacy of HBC among patients and formal health workers [43,44]. Future research should take a more in-depth consideration of the patient perspective with regards to routine tracing and its challenges.

## Conclusions

Strengthening the recording and routine use of tracing data will capitalise on this source of information for patient monitoring at the facility level and improve the accuracy of estimates of true outcomes for patients who become LTFU. This will necessitate significant investments in the health system, in the training of healthcare workers, and enhancements in data infrastructure. It is important that tracing systems and the data they generate keep abreast with evolving guidelines and rapidly shifting health service delivery.

## Paper context

Most HIV programmes in sub-Saharan Africa implement some form of routine tracing for patients who do not return for scheduled clinic visits. However, this is inconsistently done and fraught with issues resulting in many patients becoming lost to the system. We evaluated routine tracing in rural South Africa and report on gaps identified as well as offer recommendations to improve routine tracing in South Africa and similar settings.
